# Hydrogel Microparticles Functionalized with Engineered *Escherichia coli* as Living Lactam Biosensors

**DOI:** 10.3390/s19245556

**Published:** 2019-12-16

**Authors:** Conghui Ma, Jie Li, Boyin Zhang, Chenxi Liu, Jingwei Zhang, Yifan Liu

**Affiliations:** 1 Materials and Physical Biology Division, School of Physical Science and Technology, ShanghaiTech University, Shanghai 201210, China; mach@shanghaitech.edu.cn (C.M.); lijie4@shanghaitech.edu.cn (J.L.); zhangby2@shanghaitech.edu.cn (B.Z.); liuchx@shanghaitech.edu.cn (C.L.); 2 State Key Laboratory of Genetic Engineering, School of Life Sciences, Fudan University, Shanghai 200438, China

**Keywords:** whole-cell biosensor, microfluidics, hydrogel, lactam, living functional material

## Abstract

Recently there has been an increasing need for synthesizing valued chemicals through biorefineries. Lactams are an essential family of commodity chemicals widely used in the nylon industry with annual production of millions of tons. The bio-production of lactams can substantially benefit from high-throughput lactam sensing strategies for lactam producer screening. We present here a robust and living lactam biosensor that is directly compatible with high-throughput analytical means. The biosensor is a hydrogel microparticle encapsulating living microcolonies of engineered lactam-responsive *Escherichia coli*. The microparticles feature facile and ultra-high throughput manufacturing of up to 10,000,000 per hour through droplet microfluidics. We show that the biosensors can specifically detect major lactam species in a dose-dependent manner, which can be quantified using flow cytometry. The biosensor could potentially be used for high-throughput metabolic engineering of lactam biosynthesis.

## 1. Introduction

Over the years, growing environmental and economic concerns of traditional chemical industry have demanded the development of more sustainable chemical synthesis routes from renewable feedstocks. The need has been partially addressed by biorefineries, in which metabolically engineered microorganisms convert inexpensive and renewable biomass into materials of high value and usefulness [1,2,3]. Among numerous chemicals being broadly employed in the industry, lactam is an important set of compounds for the manufacture of nylon [4,5,6]. Notably, ε-caprolactam (caprolactam), featuring an annual production of ~4,000,000 tons worldwide, is extensively used in the synthesis of nylon-6 [4]. Butyrolactam is the proposed monomer of nylon-4, the most hydrophilic material in the current nylon family, and is among the C4 (four-carbon) “Top Value-Added Chemical from Biomass” reported by the US Department of Energy [7]. Recently, metabolic engineering of microbes for the biosynthesis of lactams has been demonstrated by the Keasling group [8], yet significant improvement of the pathway yield and productivity is to be made before its practical and widespread application. To optimize such engineered biological systems, synthetic biologists increasingly rely on directed evolution owing to its high efficiency [9,10]. In a typical directed evolution workflow [11], a genotypically diverse library of microorganisms is generated via techniques such as random mutagenesis (e.g., chemical and UV radiation) and gene engineering (e.g., CRISPR/Cas9) [12,13,14]. The library then undergoes a screening procedure to select variants with enhanced properties. Repeated cycles of the process can lead to microorganism variants that exhibit substantially improved performance. Currently, a major issue in directed evolution is that it is often challenging to detect the rare functional variants among a library of millions of mutants, due to which a high-throughput screening strategy is highly desired [9]. However, this has been hindered by the limited capability of sensing target products, especially small metabolite molecules such as lactams.

Lactam is a class of cyclic amides with varying ring sizes. In the lactam family, β-lactams are notable and well-studied antibiotics (e.g., penicillin), which can be specifically recognized by biological receptors (e.g., histidine kinase VbrK) [15] and detected using immunochemical techniques, such as the enzyme-linked immunosorbent assay (ELISA) [16]. The detection of other lactam species of high industrial demand (e.g., valerolactam, caprolactam and butyrolactam), however, relies largely on analytical techniques, such as liquid chromatography [17], mass [18] and Raman spectrometry [19,20], which fall short in their multiplexing capability when assaying a large number of samples. Liquid chromatography, for instance, allows the quantitative identification of lactams with high sensitivity, yet the throughput is limited to few hundreds experiments per day [21]. Therefore, it is of utmost value to develop highly scalable lactam-responsive sensors that could be operated in a high-throughput manner.

Synthetic whole-cell biosensors have recently demonstrated their great potential in environmental monitoring, biosynthesis and biotherapy [22,23,24,25]. Compared to conventional solid-state counterparts [26,27], these living sensors possess various unique characteristics, such as cost-effectiveness and renewability. A single bacterial-based whole-cell biosensor can be scaled up to millions of itself simply by over-night culture. This extremely high scalability is desired for the development of high-throughput parallel biosensing strategies where a large number of sensors are employed. Notably, Zhang and coworkers have successfully developed a lactam-responsive metabolic pathway in *Escherichia coli* (*E. coli*) [4]. The engineered bacteria system can specifically detect major lactam species at 10 mM. More recently, an artificial caprolactam-specific riboswitch has been reported as an intracellular metabolite sensor, featuring a detection limit of 50 mM [28]. Given this, a seemingly straightforward strategy to achieve high-throughput lactam sensing is to compartmentalize single biosensors to detect different targets. However, reliable sensing of a target species requires the readout from a reasonable population of the whole-cell biosensors due to their heterogeneous nature. Moreover, it is technically challenging to handle these delicate and minute (several microns) living cells. Therefore, a more robust and easy-to-handle format of such living biosensors needs to be developed before it can be practically used in high-throughput lactam producer screening.

**Figure 1:**
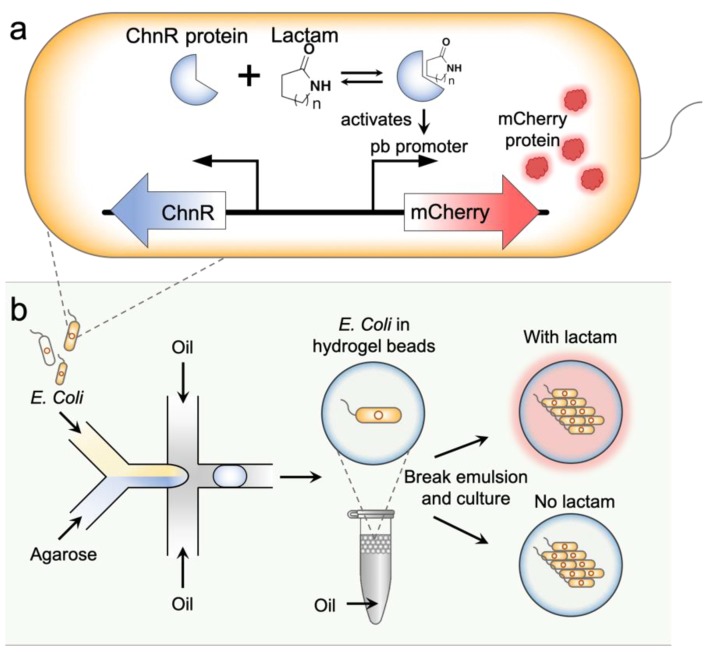
Schematic illustration of the living lactam biosensors. (**a**) The ChnR/Pb transcription factor-promoter pair-based lactam sensing pathway engineered in *E. coli*. (**b**) The hydrogel-based living biosensors generated by encapsulating engineered *E. coli*. cells into agarose microgels. The recovered microgels are further incubated to form colonies and express mCherry fluorescent protein if lactam is present in the environment, which can be fluorescently detected.

Due to their biocompatible, solution-like and non-fouling nature, hydrogel-based materials are ideal substrates for biosensing [29]. Digital formats of hydrogels, such as microparticles, can be synthesized by microfabrication means, enabling multiplexed sensing in biological samples. Encapsulating whole-cell biosensors in hydrogels have been reported by several groups [30,31]. Futra and coworkers encapsulated *Aliivibrio fischeri* in alginate microgels for monitoring heavy metal levels in environmental samples [31]. Li and coworkers demonstrated encapsulation of reporter bacteria in alginate microbeads to sense homoserine lactone [30]. In these works, however, the use of hydrogel encapsulation is solely for immobilization and the ease of handling. Continuous culturing of whole-cell biosensors in the gel is yet to be explored to prove the biosensor robustness. Moreover, high-throughput screening of the sensing behavior of such digitized biosensors was not investigated. In this paper, we report a type of living biosensor—hydrogel microparticles containing colonies of engineered bacteria—for specific and reliable lactam detection. The biosensing principle is depicted in Figure 1a. Briefly, the sensing relies on a lactam-inducible mCherry expression system built in an *E. coli* host strain [4]. In the system, a lactam responsive protein ChnR encoding gene is designed. Once the ChnR protein is expressed and bound to lactam molecules, it activates the Pb promoter that is placed next to an mCherry encoding gene, which further initiates the expression of the mCherry fluorescent protein. The living biosensors are manufactured using high-throughput droplet microfluidic techniques. As shown in Figure 1b, the lactam-responsive bacteria are co-encapsulated with melted agarose solution, forming agarose microgels containing embedded cells. The microgels are then placed in the appropriate medium for further culture. This results in colonies of hundreds of cells in each microgel, thereby significantly amplifying the fluorescent signal that can be readily detected by conventional fluorescence detection means. The usefulness of the biosensors is validated by sensing caprolactam, valerolactam and butyrolactam in a dose-dependent manner. Importantly, the embedded cells are protected by the hydrogel shell, thus more resistant to environmental influences. Moreover, these digitalized living hydrogel microparticles are directly compatible with various high-throughput digital analytical platforms, such as fluorescent-activated cell sorting (FACS), droplet fluorescence reader and various microdroplet manipulation modules, allowing up to thousands of individual biosensors to be assayed per second. We anticipate this living lactam biosensor will be useful in the development of high-throughput screening strategies for lactam bioproduction microorganisms.

## 2. Materials and Methods

### 2.1. Microfluidic Device Fabrication

The fabrication of the microfluidic droplet maker was carried out using polydimethylsiloxane (PDMS)-based soft lithography. First, to create photoresist masters, negative photoresist SU-8 3025 (MicroChem) was spin coated on a 3-inch silicon wafer (University Wafer) at 4000 rpm, which resulted in a uniform SU-8 layer of approximately 20 μm thickness. After a pre-bake of 10 min at 95 °C, the wafer was covered with a plastic photo-mask and exposed under a 120 mW UV lamp (M365L2, Thorlabs) for 2 min. The subsequent post-bake took about 10 min at 95 °C and the wafer was then developed in SU-8 developer solution (MicroChem) for ~15 min. The fabricated master was cleaned with isopropanol and ethanol, and blow-dried using a nitrogen gun. Next, the PDMS precursor (SYLGARD 184, Dow Corning) was mixed with a curing agent at a ratio of 10:1 (w:w) and poured over the master, followed by a curing process of ~3 h at 60 °C. The cured PDMS slab was peeled off from the master and the inlet/outlet ports were created using a 0.7 mm hole puncher. The slab was then bonded to a clean glass slide with the aid of oxygen plasma treatment. Before use, the fabricated devices were treated by a commercial water repellent, Aquapel (PPG Industries), to render the channel surfaces hydrophobic.

### 2.2. Bacteria Strain and Culture Cnditions

The chemicals used in this work were purchased from Sigma Aldrich unless otherwise stated. The lactam responsive *E. coli* strain JZ-439 was engineered from the commercially available strain DH10B. The host strain DH10B, genotype F−mcrA crmrr-hsdRMS-mcrBC) r-hsdRMSmcrBC) and oligonucleotide139 Δ(ara, leu) 7697 galU galK alrpsL nupG, was purchased from Life Technologies. The major gene engineering was the integration of the pBbSLactamC-mCherry biosensor system that is constructed on a single plasmid. The pBbSLactamC-mCherry plasmid contains a weak RBS 5′ of ChnR and the SC101 origin of replication. *E. coli* DH10B was transformed with pBbSLactamC-mCherry to form a biosensor strain capable of responding to exogenously added butyrolactam, valerolactam, and caprolactam. Details of the strain JZ-439, the plasmid scaffold and the cloning have been described elsewhere [4]. Cultures of *E. coli* JZ-439 were obtained through inoculated single colonies and over-night culture at 30 °C in Luria−Bertani (LB) medium containing 100 μg/mL ampicillin and 20 μg/mL kanamycin.

### 2.3. Hydrogel Microparticle Generation

The harvested *E. coli* culture was rinsed with PBS and resuspended in fresh LB medium. Cell density was calculated through measuring optical density (OD) at 600 nm using a NanoDrop spectrophotometer. The cell solution was then diluted to approximately 5 × 10^8^ cells/mL, corresponding to a final concentration of ~5 cells/microparticle. The agarose solution was prepared by melting 2% w/w ultra-low melting temperature agarose in PBS at 90 °C using a metal bath. The cell and agarose solutions were co-flowed into the microfluidic droplet maker as the discrete phase at equal flowrates of 100 μL/h using syringe pumps, whereas a fluorinated oil (HFE7500, 3M) with added 2% v/v ionic Krytox surfactant (DuPont) was pumped in at 600 μL/h as the continuous phase. During the droplet generation experiments, a desktop fan heater set at 80 °C was placed at a proper distance to the agarose solution to keep it warm. The collected droplets were kept at 4 °C to facilitate agarose gelling. The resultant agarose microparticles were released from the oil phase by adding equal volumes of 20% v/v 1H,1H,2H,2H-perfluoro-1-octanol in HFE7500 followed by rinsing with PBS (containing 0.1% Tween-20) thrice and resuspended in LB. The collected droplets and released microgels were loaded to a chambered cell counting slide (Countess, Thermo-Fisher Scientific) and observed under an inverted microscope.

### 2.4. Biosensing Protocol and Data Analysis

Stocks of caprolactam, valerolactam, butyrolactam, lysine and glutamic acid were first prepared in DI water at 20× concentrations. A total of 100 μL of agarose microgel sensors was mixed with 100 μL as-prepared chemical solution and 1.8 mL LB medium containing 0.0002% w/w arabinose. The resulted 2 mL solution was then incubated for the indicated amount of time in a shaker at 30 °C, for the colony formation and biosensing. After the incubation, the samples were filtered with 10 μm mesh-size nylon membranes to remove free bacteria in the solution and then kept on ice. In the biosensor calibration experiments, the microgels were monitored under a fluorescence microscope at indicated time spots to obtain the growth curve. Representative micrographs were taken and the fluorescent intensity of microgel beads was analyzed using the computer software Image J. In the dose-response experiments, microgels were analyzed using a FACS-based single-cell sorter (Namocell). To filter out empty microgel beads, an FSC lower threshold of 300 was used in the setting. The incubation time for all the experiments was fixed to 16 h, except for further indication. The fluorescence intensity from caprolactam, valerolactam and butyrolactam FACS datasets were directly used for plotting biosensing behavior without further adjustments.

## 3. Results

**Figure 2:**
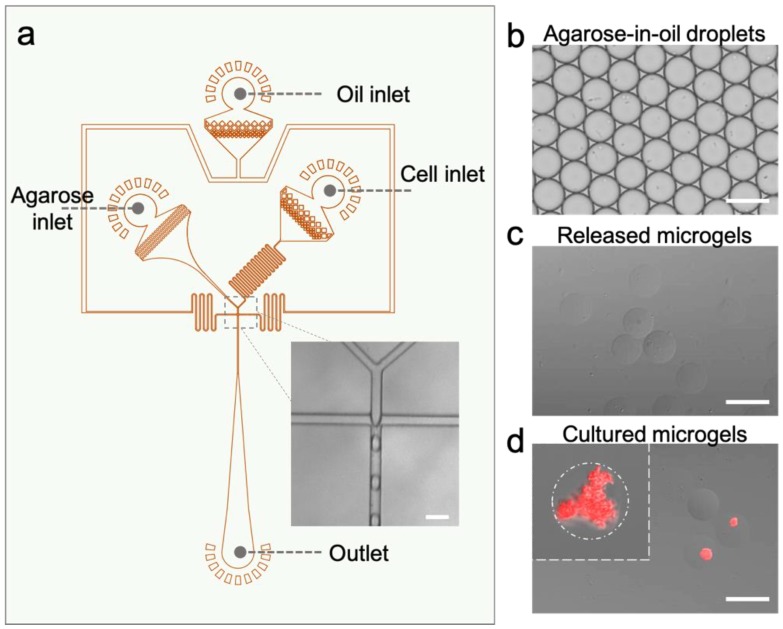
Microfluidic manufacturing of the living lactam biosensors. (**a**) The layout of the droplet microfluidic device for the generation of agarose microgels with encapsulated engineered *E. coli* cells. (**b**,**c**) Microscopic images of (**b**) the as-generated agarose-in-oil droplets and (**c**) the released microgel beads suspended in an aqueous solution. (**d**) Stacked fluorescent micrograph showing microcolonies expressing mCherry proteins in the microgels induced by 50 mM caprolactam. Scale bars: 50 µm.

The layout of the microfluidic device for hydrogel microparticle generation is shown in Figure 2a. The device is a modified flow-focusing dropmaker featuring two distinct aqueous inlet channels for cell suspension and agarose solution, respectively. Such a coflow geometry separates the two flow streams until they meet right before the flow focusing junction, avoiding prolonged contact of cells with molten agarose (~60 °C) that may affect cell viability. The junction was designed to be 25 µm wide, suitable for generating ~30 µm droplets. In the experiments, the flowrate ratio was kept to 1:3 for the discrete/continuous phase so that the droplet breakup was under the dripping regime (Figure 2a inset) to ensure high monodispersity [32]. The generated agarose droplets are shown in Figure 2b, having diameters of 34.04 ± 0.97 µm (*n* = 548) and a coefficient of variation of 0.028. Given an overall aqueous flowrate of 200 μL/h, the droplet generation rate was estimated to be ~2690 droplets per second. It can be clearly seen that *E. coli* cells were encapsulated in the droplets. The droplets were well organized because they form a self-assembly layer on top of the oil (density = 1.61 g/mL). The self-assembly was arranged into a hexagonally ordered structure, topologically similar to colloidal crystals [33]. Figure 2c displays agarose microgel beads that were released in PBS buffer after gelling. The cured beads exhibited no noticeable change in morphology and size. Due to reduced discrepancy in the reflective index, the microgel beads were less visible in an aqueous solution than in oil. To test whether the *E. coli* cells can survive in the hydrogel through all the microfluidic steps and whether they are responsive to lactam, we further cultured the collected beads in LB buffer with the presence of 50 mM caprolactam. After 16 h of culturing, it was found that the agarose microgels maintained their shapes, while the encapsulated *E. coli* cells reproduced to colonies that occupied a noticeable portion of the gel beads (Figure 2d). Importantly, the expression of mCherry fluorescent protein was confirmed (red color of the colonies), suggesting that the cells indeed responded to the present lactam level. It should be noted that ~39% (*n* = 75) of the incubated microgel beads contained *E. coli* colonies, which was lower than the estimated value of more than 90% according to the Poisson distribution for the given cell loading concentration. This was possibly due to cell death or leaking during the encapsulation and subsequent handling steps. Increasing the agarose concentration might prevent the cell escaping from the agarose matrix, yet it was found that concentrated agarose gel possessed an increased inhibition effect on cell growth; the *E. coli* cells could barely grow and replicate in 1.5% w/w agarose (data not shown), possibly owing to enhanced physical constriction. It should be noted that, although only 39% of the as-generated gel beads can be analyzed for their lactam sensing behavior, the effective generation rate is still reasonably high (~1000 per second). That means, for typical FACS assays where a few millions of analytes are required, the sample can be prepared in one hour. Overall, these results verify that functional hydrogel microparticles can be conveniently generated at a high throughput using our droplet microfluidic strategy.

**Figure 3:**
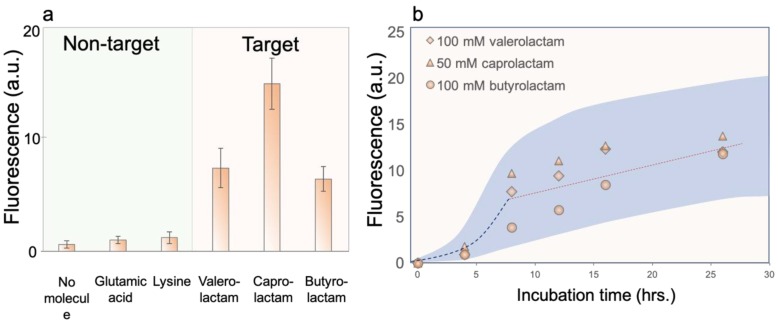
Biosensor characterization. (**a**) Biosensor response to non-target and target chemicals. The concentration of all the chemicals are kept to 50 mM. The symbols and error bars refer to the mean and standard deviation of the values from separate microgel sensors (*n* = 400). (**b**) Influence of incubation time on the biosensing behavior of different target lactam species. The dash guidelines highlight the exponential and saturation regime, respectively. The shadowed area represents the extent of standard deviation of all data sets.

The living hydrogel microparticles were expected to be readily functional upon formation. To test whether the biosensors can specifically response to lactam species, we performed a series of control experiments. The as-fabricated microgel beads were co-incubated with equal molar concentration (50 mM) of two non-targets, glutamic acid and lysine, which are typical lactam precursor intermediates, and three major lactam molecules, valerolactam, caprolactam and butyrolactam. The incubation times were all kept to 16 h and the fluorescent intensity of representative microgel beads (*n* > 50) in each experiment were plotted in Figure 3a. As shown, the non-target glutamic acid and lysine only led to a slight increase in the fluorescence as compared to the negative control where no molecules were added, while all the target lactam species contributed to significantly enhanced fluorescence. Caprolactam induced the highest signal among the three different lactam species, ~92.9-fold of the background (no molecule), in agreement with previous reports [4]. Valerolactam and butyrolactam resulted in a ~43.4- and 34.5-fold increase in the fluorescence, respectively. Compared to these lactam targets, the signals caused by non-target molecules (<6-fold) can be neglected, verifying that the biosensors exhibited reasonably good selectivity of lactam against other non-target chemicals.

As the cells further grew in the hydrogel during the incubation process, the sensing processes terminated at different incubation time stages might lead to vastly different signals. Therefore, all the sensing readouts must be recorded at a fixed duration when the signal amplification curves reach saturation. In order to obtain the desired incubation time, we moved to investigate the effect of the duration of incubation on the sensing behavior. In the experiments, high lactam concentrations (100 mM) were used to ensure high signal amplification. The concentration of caprolactam was intentionally lowered to 50 mM as it was previously reported that caprolactam possesses high toxicity to *E. coli* cells when exceeding 50 mM [4]. Figure 3b plots sensing behavior for three different lactam species measured at distinct time spots. As highlighted by the dashed trend lines, the signals of valerolactam and caprolactam experienced an exponential increase before turning into a plateau stage (at ~8 h) where the fluorescence no longer exhibited significant changes with time. Such a tendency suggests an interesting amplification characteristic of *E. coli* cells compartmentalized in limited space (microgel beads in our case): (1) exponential amplification of *E. coli* cells at early stages, alike bulk culture; and (2) self-limited amplification due to the constraint of hydrogels once the *E. coli* colonies occupied a considerable portion of the microgel. Interestingly, the intensity of 100 mM butyrolactam was found to be increased linearly up to 25 h. In our biosensing system, the ChnR transcription factor naturally respond to cyclohexanone, a six-member ring lactone compound [4]. Valerolactam and caprolactam are structurally more similar to cyclohexanone than butyrolactam. Therefore, these two compounds trigger transcriptional activation within a shorter time frame and allow the biosensor to reach full induction by 8 h. Another potential factor is that valerolactam and caprolactam are slightly more hydrophobic and could permeate across a membrane easier. Again, it was found that caprolactam led to a most noticeable amplification in the fluorescence signal, although its concentration was halved. The incubation time of 16 h was determined to be used in the sensing experiments.

**Figure 4:**
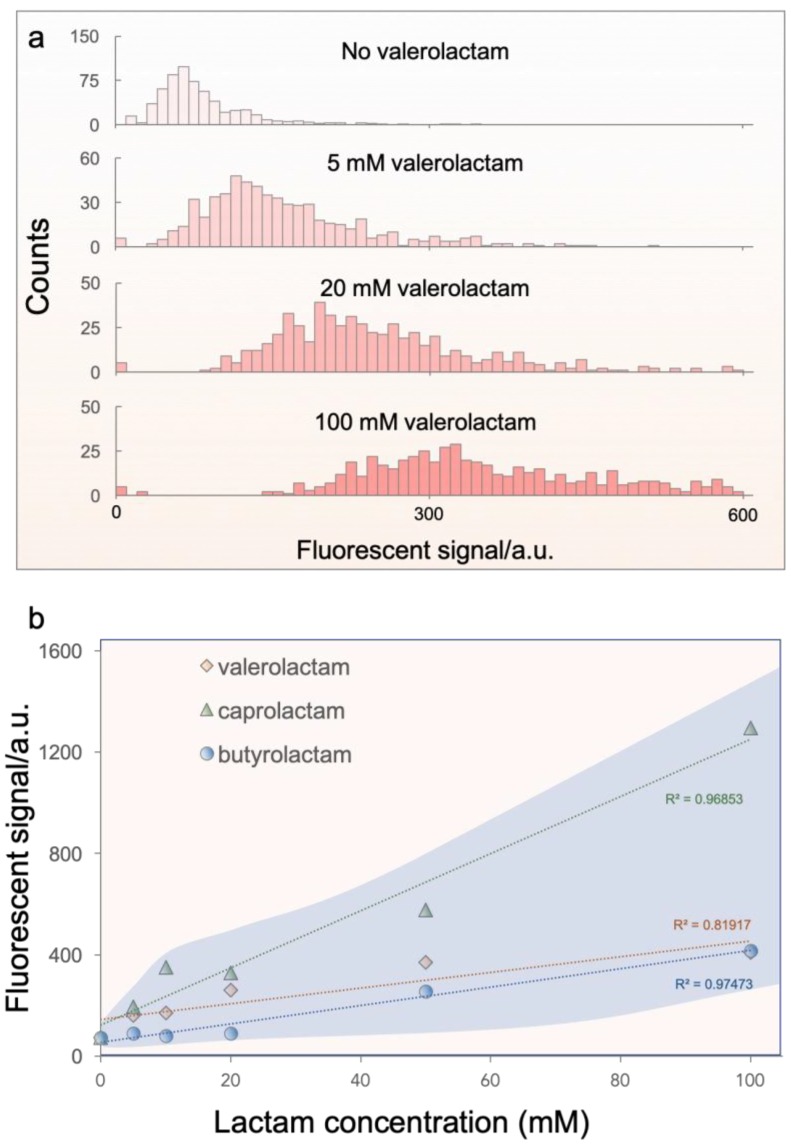
Biosensor calibration for dose-dependent lactam detection. (**a**) Fluorescent-activated cell sorting (FACS) histograms of the fluorescence intensity of individual microgel sensors subject to various levels of valerolactam. Each histogram collects the data from 600 microgels. (**b**) Dose-response plot of lactam biosensing. The dash guidelines highlight the linear trend of the dose-response relations. The shadowed area represents the extent of standard deviation of all data sets.

Due to the digitalized nature, the living lactam biosensors should be directly compatible with high-throughput cell analysis tools, such as FACS, for signal detection. To assess the feasibility of the microgel beads being analyzed by such means, we performed FACS on the microgels co-incubated with various lactam levels. It turned out that our microgels can be conveniently assayed by FACS. Figure 4a shows the histograms of microgels subject to increasing concentrations of valerolactam. Each histogram collects the fluorescent signal from 600 different microgel beads. As can be seen, the signal distributions were gradually shifted to the right side along with the increase of the valerolactam dose. To better visualize the trend, we plotted the dose-response relations of three major lactam species in Figure 4b. Here, the fluorescent signals were obtained from FACS assays on individual microgels (*n* = 600) subject to various levels of valerolactam, butyrolactam and caprolactam. Fairly linear correlations between fluorescence signals and target concentrations could be observed for all the lactam species. At the concentration of 5 mM, valerolactam and caprolactam could be routinely detected, having a ~2.2- and 2.6-fold increase in fluorescence, respectively. Butyrolactam featured a rather high limit of detection, ~ 50 mM. The sensing of caprolactam exhibited the largest dynamic range, a 7.8-fold increase at 100 mM as compared to the baseline signal. Such a dynamic range is about 3.8 times better than the previous reported lactam sensor based on discrete *E. coli* cells, which exhibited a dynamic range of 2.08 [4]. Moreover, it is worthwhile to note that the reported toxicity of high concentration caprolactam (>50 mM) did not affect the sensing behavior of our living biosensors, which suggests a potential protection effect of the hydrogel shell to the encapsulated cells [4]. The exact mechanism of the protect effect is to be further investigated. These results validate that our living lactam biosensors are capable of detecting different lactam species in a dose-responsive fashion.

## 4. Discussion

It is obvious that the presented living hydrogel biosensors do not surpass conventional platforms, such as mass spectrometry, in terms of limit of detection and response time. However, their minute size (~30 μm) allows them to be used in an extremely confined microenvironment (e.g., a droplet) to continuously monitor lactam levels. Moreover, the limit of detection of the biosensor can be significantly lowered through the integration of genetic cascades [34]. Currently, high yield biosynthetic strains for butyrolactam, valerolactam and caprolactam produces around 17.6 mM, 7 mM and 17 μM, respectively [4]. When the producer strains are confined in limited environments (e.g., droplets), the local concentration may be even higher. Our biosensor’s dose-dependence behavior was characterized to be between 0–100 mM, and thus can be readily utilized to screen lactam butyrolactam and valerolactam production. Furthermore, up to 10,000,000 of the hydrogel sensors can be facilely fabricated in 1 h. The biosensors can be stored at 4 °C or possibly −80 °C with the addition of glycerol. However, immediate use is favored as prolonged storage may lead to cell death and thus degraded biosensing performance [31]. Benefiting from these unique features, the presented lactam biosensor can be potentially useful in directed evolution strategies where millions of bio-producer and biosensor pairs are separately compartmentalized for parallel ultra-high throughput screening. Such a screening methodology is currently under development and will be reported in due course.

In conclusion, we have presented a novel type of living-cell-based biosensors for specific lactam detection. The biosensor is composed of agarose gel microparticles and encapsulated *E. coli* cells that are engineered to be responsive to lactam. The sensor specifically detects major lactam species of great industrial demand, namely valerolactam, caprolactam and butyrolactam, with improved dynamic range compared to discrete whole-cell biosensors. Importantly, the sensors are generated in extremely high throughput with the use of droplet microfluidics. The digital nature of such hydrogel microparticle-based biosensors enables quantitative monitoring of their sensing behavior. Given its robustness, miniaturized size and facile fabrication, the presented living hydrogel lactam sensors can potentially pave the way for ultra-high throughput bio-screening. Investigations are currently underway for a high-throughput bio-screening strategy incorporating droplet microfluidics and the biosensors developed.
